# Potential Biosorbent Derived from *Calligonum polygonoides* for Removal of Methylene Blue Dye from Aqueous Solution

**DOI:** 10.1155/2015/562693

**Published:** 2015-01-15

**Authors:** Asma Nasrullah, Hizbullah Khan, Amir Sada Khan, Zakaria Man, Nawshad Muhammad, Muhammad Irfan Khan, Naser M. Abd El-Salam

**Affiliations:** ^1^Department of Chemistry, University of Science and Technology, Bannu, Khyber Pakhtunkhwa 28100, Pakistan; ^2^PETRONAS Ionic Liquid Centre, Department of Chemical Engineering, Universiti Teknologi PETRONAS (UTP), 31750 Tronoh, Perak, Malaysia; ^3^Interdisciplinary Research Centre in Biomedical Materials (IRCBM), COMSATS Institute of Information Technology, Lahore, Pakistan; ^4^Department of Chemical Engineering, Universiti Teknologi PETRONAS (UTP), 31750 Tronoh, Perak, Malaysia; ^5^Riyadh Community College, King Saud University, Riyadh 11437, Saudi Arabia

## Abstract

The ash of *C. polygonoides* (locally called balanza) was collected from Lakki Marwat, Khyber Pakhtunkhwa, Pakistan, and was utilized as biosorbent for methylene blue (MB) removal from aqueous solution. The ash was used as biosorbent without any physical or chemical treatment. The biosorbent was characterized by using various techniques such as Fourier transform infrared spectroscopy (FTIR), thermogravimetric analysis (TGA), and scanning electron microscopy (SEM). The particle size and surface area were measured using particle size analyzer and Brunauer-Emmett-Teller equation (BET), respectively. The SEM and BET results expressed that the adsorbent has porous nature. Effects of various conditions such as initial concentration of methylene blue (MB), initial pH, contact time, dosage of biosorbent, and stirring rate were also investigated for the adsorption process. The rate of the adsorption of MB on biomass sample was fast, and equilibrium has been achieved within 1 hour. The kinetics of MB adsorption on biosorbent was studied by pseudo-first- and pseudo-second-order kinetic models and the pseudo-second-order has better mathematical fit with correlation coefficient value (*R* ^2^) of 0.999. The study revealed that *C. polygonoides* ash proved to be an effective, alternative, inexpensive, and environmentally benign biosorbent for MB removal from aqueous solution.

## 1. Introduction

Environmental pollution is a serious and challenging problem all over the world because of the rapid progress in society, science, technology, and industries. The industrial effluent containing both inorganic and organic toxic material which discharging into surface water that seriously affect biodiversity, ecosystem functioning and natural activities of aquatic system. Among these pollutants one such pollutant is the synthetic dyes which are considered to be the most common and toxic water pollutants [1–5]. The dye compounds have been extensively used in various industries to colorize the products, such as in papers and pulp, textiles, plastics, wool, paints, rubber manufacturing, printing, cottons, cosmetic products, foods, and pharmaceuticals. The extensive use of dyes and dyes containing compounds disturb the aquatic system by inhibiting the sunlight from reaching water and thus reducing photosynthesis and increasing the biological oxygen demand (BOD) and chemical oxygen demand (COD) values [1–3, 5]. Among these dyes some cause depletion of the dissolved oxygen content of water thus affecting aquatic life badly. In addition, certain textile dyes are carcinogenic and toxic to living organisms and have adverse effect on human health, domestic animals, and wildlife [6].

Among the dyeing agents, methylene blue (MB) which is a heterocyclic aromatic chemical compound is widely used in the textile industries [6–9]. MB and MB like other textile dyes can cause eye burns in humans and animals, skin irritation, dyspnea, convulsions, cyanosis, tachycardia, and dyspnea, and if ingested can cause gastrointestinal tract irritation, nausea, vomiting, also diarrhea, and so forth [10–12]. Due to these toxicological and hazardous effects of dyes on environment and subsequently on living organism, the removal of these dyes from wastewater is a key challenging task for researchers and an important area of research directed towards a better life [10, 13–15].

There are various physical and chemical methods used for removal of textile dyes from aqueous media. These include ion exchange, membrane filtration, electrochemical destruction, ozonation, flotation, chemical coagulation, biological and chemical oxidation, precipitation, and electrokinetic and adsorption methods [16–19]. However most of these methods have some major drawbacks, such as the applicability at relative high concentration of dye, insufficient dye removal, high cost and production of extra waste [20]. Similarly, the biological methods often demand a very strict control of experimental conditions and especially the pH and the decontamination process is usually very slow. Alike, in chemical oxidation methods, the biodegradation products are often carcinogenic and toxic in nature which affects the aquatic life.

Activated carbon has been extensively used as an adsorbent for the removal of dyes because of the acidic nature and showed pores nature of its surface [21–23]. However, the activated carbon is costly and challenging in regeneration which raises the cost of waste water treatment [24, 25]. Thus, there is a great demand for such type of adsorbent which is cheaper and still has high adsorption capability towards pollutants and dyes without any additional expensive pretreatment. Presently cellulose and lignocellulosic biomass have got considerable attraction because of the abundance, effectiveness, low cost, and environmentally friendly nature of these biopolymers. Thus biosorption has been proved to be the most effective technique for the removal of MB from the aqueous solution [16, 26–29].

In this research work* C. polygonoides* (locally called balanza, which is used as fuel for domestic cooking purposes and in bricks kiln industry) was collected from Lakki Marwat, Khyber Pakhtunkhwa, Pakistan, and utilized as biosorbent for MB removal from aqueous solution without any physical or chemical treatment. The utilization of this alternative, abundant, low cost, and environment friendly bioadsorbent will effectively reduce both the waste disposal problem and the cost of waste decontamination.

## 2. Materials and Methods

### 2.1. Methylene Blue

All the chemicals used in the present research work were of analytical grade and purchased from Sigma-Aldrich, BDH, and Merck. MB with chemical formula C_16_H_18_ClN_3_S_3_H_2_O (Table 1) was used as model adsorbent to study the adsorption capacity of biosorbent. The stock solution of 1000 ppm of MB was prepared and subsequently their solutions of desired concentration were prepared by applying the dilution formula (*M*_1_ *V*_1_ = *M*_2_ *V*_2_).

### 2.2. Preparation of Biosorbent

**Figure 1:**
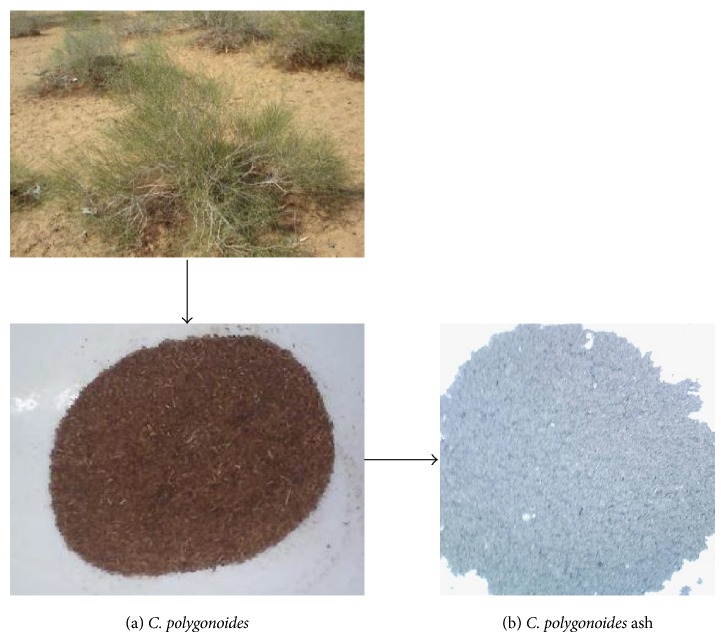
Digital photographs of* C. polygonoides* and its ash.

The* Calligonum polygonoides* ash was washed with double distilled water to remove soluble impurities and then dried in oven at 110°C for 5 hours. The powdered material thus obtained was sieved through 45 *μ*m sieve using Retsch AS 200 basic and was stored in air tight glass bottle for further experimental study (Figure 1).

### 2.3. Characterization of Biosorbent

#### 2.3.1. Scanning Electronic Microscopic (SEM) Analysis

The surface structure of biosorbent was analyzed using scanning electron microscopy (JMT-300, JEOL).

#### 2.3.2. Surface Area Analysis

The surface area of* C. polygonoides* ash sample was determined using Brunauer-Emmett-Teller (BET) method and the pore size diameter was obtained by BJH method from the adsorption/desorption isotherm of nitrogen gas, at 77.0 K, employing Micromeritics ASAP 2010 apparatus.

#### 2.3.3. Fourier Transform Infrared Spectroscopy

The chemical structure and nature of functional groups* C. polygonoides* ash were studied using FTIR transmission spectra on a Perkin Elmer Spectrum One. For FTIR spectra measurement, sample was mixed with KBr in the ratio of 1/1000 and pressed into the pellet using a Perkin Elmer hydraulic pump. FTIR spectrum was recorded in the wave number range from 4000 to 450 cm^−1^ with resolution of 5 cm^−1^.

#### 2.3.4. TGA Analysis

To investigate the thermal characteristic or weight-loss profiles of* C. polygonoides* ash, a thermogravimetric analyzer (Pyris-1, Perkin Elmer, Shelton, CT) was used over the temperature range from 50°C to 800°C at heating rate 10°C/min. The analysis was conducted under N_2_ gas with flow rate of 50 mL/min and weight of samples taken was around 5–7 mg.

#### 2.3.5. Particle Size Analysis

The particle size distributions were measured using Mastersizer 2000 ver. 5.54, serial number: Mal 18486 (Malvern Ltd., UK). For particles size distribution measurement, 0.5 g of* C. polygonoides* ash was loaded into the tray and dispersed by compress air to bring sample into laser beam for measurement of the particle size distributions.

### 2.4. Experiments for Dye Adsorption

The biosorption experiment of MB was conducted by adding 100 mg of biosorbent to 25 mL of aqueous solution of MB dye (4–10 mg/L) in 50 mL of conical flask and shaken at 300 rpm in orbital shaker at 298 K. After specific interval of time, sample aliquots were withdrawn and centrifuged to separate the dye loaded from dye solution. The remaining concentration of MB was measured spectrophotometrically by measuring the absorbance of the supernatant (dye solution) left after centrifugation. To find the optimum experimental conditions for MB removal, for MB removal, the effect of time (10–120 min), initial dye concentration (2 mg/L–10 mg/L), biosorbent loading (20–100 mg), and shaking velocity (100–600 rpm) were thoroughly studied.

The color removal efficiency (*R*) and adsorption capacity of* C. polygonoides* ash were measured by applying the following equations:

(1) $$\begin{array}{c} \left(R\%\right)=\left(\frac{C_{0}-C_{t}}{C_{0}}\right)100 \end{array}$$

(2) $$\begin{array}{c} q_{t}=\frac{\left(C_{0}-C_{t}\right)V}{m} \end{array}$$

(3) $$\begin{array}{c} q_{e}=\frac{\left(C_{0}-C_{e}\right)V}{m}, \end{array}$$

where *q*_*e*_ and *q*_*t*_ (mg/g) are the amount of MB adsorbed at time *t* and equilibrium, respectively; *C*_0_, *C*_*t*_, and *C*_*e*_ (mg/L) are concentration of MB at initial, time *t*, and equilibrium, respectively; *V* is the solution volume and *m* (g) is the mass of biosorbent.

### 2.5. Kinetic Study and Model Fitting

To study the adsorption mechanism of MB on biosorbent pseudo-first- and pseudo-second-order kinetic models were used.

#### 2.5.1. The Pseudo-First-Order Equation

The pseudo-first-order kinetic model or Lagergren kinetic equation is generally expressed as follows:

(4) $$\begin{array}{c} \log\left(q_{e}-q_{t}\right)=\log q_{1}-k_{1}\cdot \frac{t}{2.303}, \end{array}$$

where *q*_*e*_ is the amounts adsorbed (mg/g) at equilibrium, *q*_*t*_ is the amounts adsorbed (mg/g) at any time, and *k*_1_ is the adsorption rate constant for pseudo-first-order (s^−1^).

#### 2.5.2. The Pseudo-Second-Order Model

If the rate of sorption is a second-order mechanism, the pseudo-second-order kinetic rate equation is used and it is expressed as

(5) $$\begin{array}{c} \frac{dqt}{dt}=k_{2}{\left(q_{2}-q_{t}\right)}^{2}\\ \frac{{dq}_{t}}{{\left(q_{2}-q_{t}\right)}^{2}}=k_{2}dt. \end{array}$$

By integration of (3) for the boundary conditions when *t* = 0 and *q*_*t*_ = *q*_*i*_

(6) $$\begin{array}{c} \frac{1}{q_{2}-q_{t}}=\frac{1}{q_{2}}+k_{2}dt, \end{array}$$

where *k*_2_ (g/mg*·*min) is the rate constant of pseudo-second-order:

(7) $$\begin{array}{c} \frac{t}{q_{t}}=\frac{1}{k_{2}q_{2}^{2}}+\frac{1}{q_{2}}t. \end{array}$$

If *t*/*q*_*t*_ is plotted against *t*, it gives a straight line, which means that the adsorption follows pseudo-second-order kinetics. This model is based upon the assumption, if the rate limiting step may be chemisorption which involve valence forces due to the electron sharing or exchange of electron between the adsorbent and adsorbate.

## 3. Results and Discussion

### 3.1. Particle Size Distributions

After thermal treatment, the particle size of adsorbent decreases. Decrease in particles size of adsorbent usually results in enhanced adsorption of dye because the adsorption capacity of the adsorbent is directly proportional to the exposed surface area and inversely related to the particle diameter of nonporous material [42]. By increasing the particle size, the number of particles per given mass decreases which results in decrease in specific surface area and hence the adsorption capacity. The particle size distribution of pure* C. polygonoides* powder changed from (0.1) 12.39 *μ*m, (0.5) 120.79 *μ*m, and (0.9) 480.309 *μ*m to (0.1) 2.36 *μ*m, (0.5) 27.90 *μ*m, and (0.9) 382.84 *μ*m, respectively, after burning. The* C. polygonoides* ash sample becomes black in color with some gray particles, resulting from different stages of carbon combustion during burning process of the biomass. The amount of black particles decreases with increasing the calcination time and temperature.

### 3.2. Surface Area (BET)

The process of adsorption is a multistep complex phenomenon and therefore many factors affect the phenomenon. Among these factors, the pore size of adsorbent significantly affects the adsorption process. Pores size is generally classified into various groups such as micropores (<2 nm diameter), mesopores (2–50 nm), and macropores (>50 nm). The surface chemistry of adsorbent and its pores structure considerably affect the adsorption of big molecules like MB into its structure. MB has molecular cross-sectional diameter of about 0.8 nm and cannot easily penetrate material with pores smaller than 1.3 nm. The surface area of* C. polygonoides* ash depends on the amorphous carbon content. During the dye adsorption process, the diffusion of dye molecules to the active sites takes place first, which is followed by attachment of these dye molecules to the active sites. The pore size and total pore volume thus play a decisive role in the adsorption process.

*C. polygonoides* ash has significant surface area and wide pore size distribution. The BET surface area of* C. polygonoides* ash is 4.3810 m^2^/g, whereas BJH adsorption/desorption surface area of pores of* C. polygonoides* ash is 4.119/4.7108 m^2^/g. For* C. polygonoides* ash the single point total pore volume of pores (*d*_*C*. *polygonoides*_ < 3166 Å) is found to be 0.016691 cm^3^/g. The cumulative adsorption/desorption pore volume of the pores (17 Å < *d* < 3000 Å) of* C. polygonoides* ash is 0.019823/.019556 cm^3^/g (Table 2). The* C. polygonoides* ash, thus, is found to consist of mesopores predominantly. This is what is desirable for the liquid phase adsorptive removal of metal ions and dyes.

### 3.3. Fourier Transform Infrared Spectroscopy

**Figure 2:**
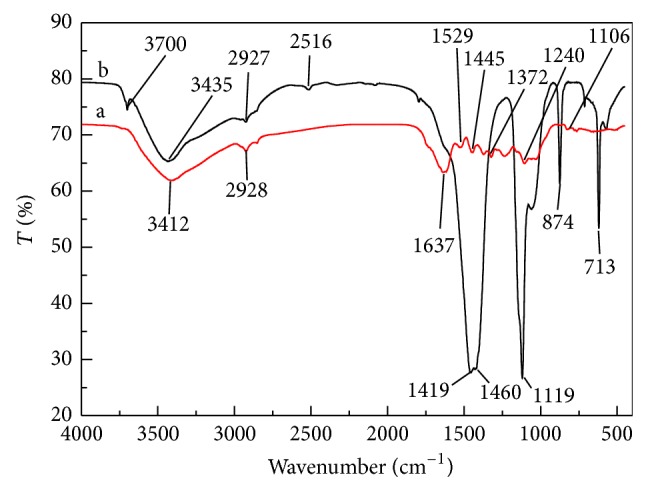
FTIR spectra of (a)* C. polygonoides* and (b)* C. polygonoides* ash.

It is important to know the exact chemical structure of the adsorbent in order to understand the adsorption process. FTIR spectroscopy was thus employed to characterize the chemical structure of* C. polygonoides* before and after thermal treatment (Figure 2). The broad band around 3400 cm^−1^ can be assigned to the stretching vibration of O–H and N–H groups. This peak is broad because of the complex vibrational modes due to participation of –OH group in hydrogen bonding. This peak represents the presence of hydroxyl group and chemisorbed water in the adsorbent [43, 44]. Beside this, the vibrational modes in this area also correspond to inter- and intramolecular hydrogen bonding. The presence of a peak at 2910 cm^−1^ shows the symmetric and asymmetric C–H stretching due to the existence of methyl and/or methylene groups [45]. The peak located at 1380 cm^−1^ is indicative of –CH_3_ group. The sharp intense peak at about 1637 cm^−1^ corresponds to O–H bending vibration of secondary adsorbed water molecules. The bands at 1528 and 1445 cm^−1^ confirm the presence of C=C bond of alkene and aromatic ring [46]. The FTIR spectra showed the presence of another peak at 1100 cm^−1^ which may tentatively be assigned to Si–O–Si and –C–O–H stretching and –OH deformation. The absorption peaks in region from 1000 to 1200 cm^−1^ represented the main skeleton of cellulose. The presence of peak at 1035 cm^−1^ represents stretching vibration of cellulose/hemicellulose and ary-OH group in lignin. The presence of polar groups on the surface is likely to give considerable cation exchange capacity to the adsorbents. The occurrence of peaks at about 793 shows the existence of Si–H [35]. The presence of adsorption band in region from 1300 to 900 cm^−1^ represents the carbonyl component (i.e., alcohols, esters, carboxylic acid, or ethers) [16, 47]. The presence of absorbance peaks in region from 900 cm^−1^ shows O–H stretching vibrations which represented aromatic groups. In* C. polygonoides* ash, the peak (900 cm^−1^) of secondary adsorbed water is not present because of thermal treatment. The appearance of peak at 3700 cm^−1^ in* C. polygonoides* ash is related to stretching of free O–H functional group [35]. The presence of polar groups on the surface is likely to give considerable cation exchange capacity to the adsorbents.

### 3.4. Morphology of Adsorbent

**Figure 3:**
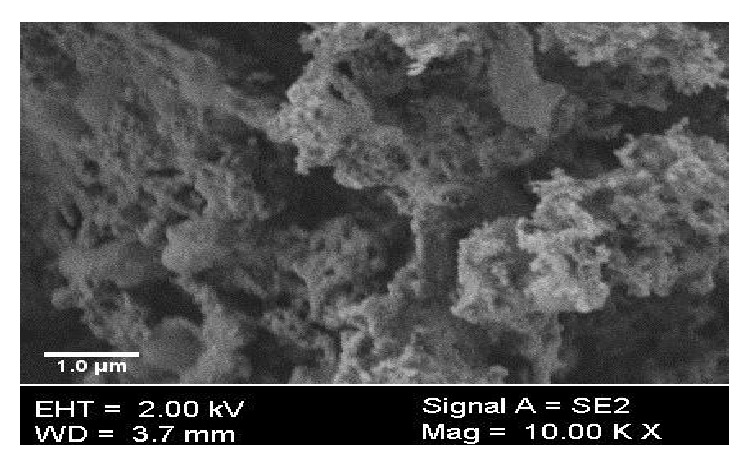
SEM analysis of* C. polygonoides* ash.

Scanning electronic microscopy (SEM) study is one the most popular, primary, and widely used characterization techniques applied for the study of surface properties and morphology of biosorbent material. Moreover, SEM study also tells about porosity and texture of biosorbent material [25, 44, 48, 49].  Figure 3 shows that* C. polygonoides* ash has small cavities on surface and has a porous texture that may provide large surface for the adsorption of the dye molecules.

### 3.5. Thermogravimetric Analysis

Thermogravimetric analysis (TGA) is an important analytical technique used to study the thermal characteristics of carbonaceous materials. The TGA provide information on the degradation process of material occurring at different temperatures and under different atmosphere.

**Figure 4:**
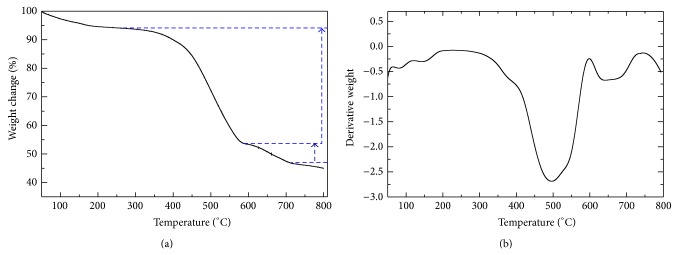
(a) TGA curve of* C. polygonoides* ash. (b) DTA curve of* C. polygonoides* ash.

The* C. polygonoides* ash was subjected to TGA under argon atmosphere. Figures 4(a) and 4(b) show the TGA and differential thermal analysis (DTA) curves for* C. polygonoides* ash, respectively. TGA result of* C. polygonoides* ash shows a typical three-stage mass loss.

- The first mass decomposition occurred below 100°C, which could attribute to water elimination/desorption which are physically absorbed in* C. polygonoides* ash. The DTA graph also shows an endothermic peak at 100°C. In this region the loss of very small amount of volatile compounds may also be contributed to the weight loss.
- The second mass loss which appeared around 300°C is due to the thermal decomposition of cellulose/hemicellulose/lignin degradation.
- The third mass decomposition occurred between 350 and 550°C, corresponding to the burning of carbonaceous residues [50].

## 4. Optimizations of Parameters

### 4.1. Effect of Contact Time and Initial Concentration of Dye

**Figure 5:**
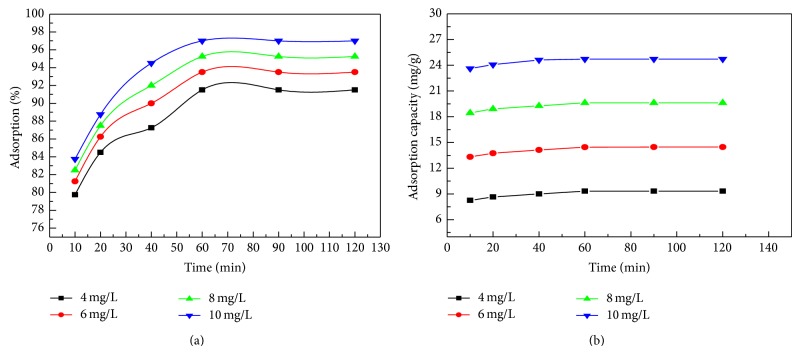
(a) Effect of contact time on adsorption of MB on* C. polygonoides* ash. (b) Adsorption capacity plot for adsorption of various concentration of MB on* C. polygonoides* ash.

The time of contact between the dye and adsorbent and also the concentration of dye can affect the adsorption process. Figures 5(a) and 5(b) show adsorption with respect to contact time and initial concentration of MB on the surface of* C. polygonoides* ash, respectively. The % removal efficiency and adsorption capacity of MB increased with increase of contact time and reached equilibrium after 60 min. Increase in contact time after 60 min cannot enhance the adsorption of MB on* C. polygonoides* ash [50]. In the beginning, the % removal of dye is very rapid due to the adsorption of more molecules of dye on the unsaturated external surface of adsorbent. After 60 min the surface pores of adsorbent are covered and it becomes difficult for dyes molecule to enter into the interior of adsorbent. The initial rapid % removal of dye may be due presence of more binding sites for adsorption of dye molecules and the slow removal of dye in the last stages may be due to occupation/saturation of these binding sites with dyes molecules [51]. The equilibrium time required for the adsorption of different dye concentration is independent of their initial concentration. The result shows that for all of the initial concentration the equilibrium was reached in the same time. Khattri and Sing also obtained the same observation during adsorption of MG on neem sawdust [35, 44, 50]. It was found that with increase of the concentration of dye from 4 to 10 mg/L the rate of adsorption and thus % removal and adsorption capacity increase. It is very common practice in the adsorption of dyes molecules that with increase of initial concentration of dyes the capacity of adsorption increases because of strong driving force and transfers of more dye molecules from aqueous phase to solid phase increase [15].

Tables 3(a) and 3(b) show a comparison of % removal and adsorption capacity of* C. polygonoides* ash with that of other adsorbents reported in the literature. It becomes clear that the adsorption capacity of the* C. polygonoides* ash is higher than many of the reported adsorbents.

### 4.2. Effect of Shaking Speed

**Figure 6:**
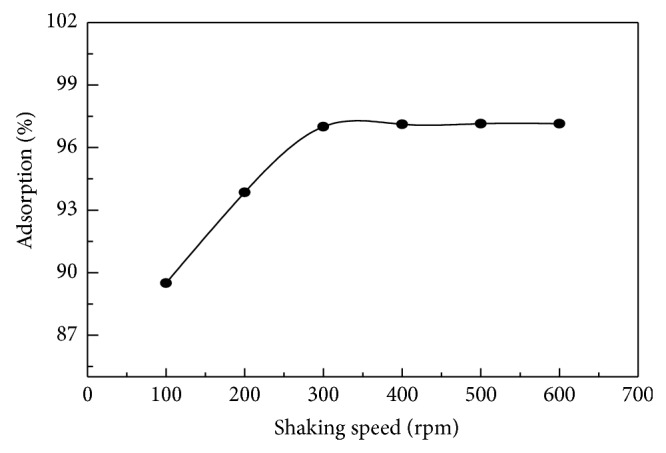
Effect of shaking speed on adsorption of MB on* C. polygonoides* ash.

To investigate the effect of stirring speed on the adsorption of MB on* C. polygonoides* ash, the experiments were carried using different stirring speed from 100 to 600 using dye concentration of 10 mg/L, contact time was 60 min, and the temperature was 298 K.  Figure 6 shows the effect of shaking speed on the MB adsorption. With increasing of the shaking speed the adsorption or % removal of dye from aqueous solution was increased. This increase in adsorption reached a maximum at 300 rpm and after this there is no such considerable increase in the adsorption of dyes. This increase in adsorption reached a maximum at 300 rpm and, further increase in speed had no significant effect on the adsorption of the dye. The increase in adsorption of MB may be to the decrease in the thickness of diffuse layer around the surface of adsorbent with increasing the stirring speed [13].

### 4.3. Effect of Adsorbent Dose

**Figure 7:**
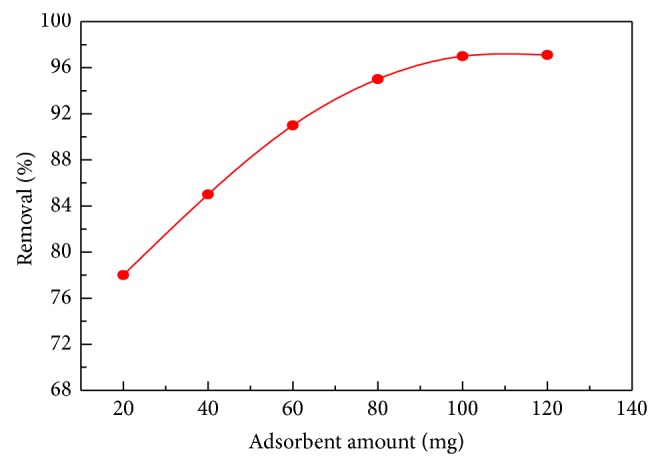
Effect of adsorption dose on MB adsorption on* C. polygonoides* ash.

The amount of adsorbent greatly affects the removal of dye from aqueous solution. The effect of* C. polygonoides* ash amount on MB adsorption was investigated in the range from 20 mg to 100 mg.  Figure 7 shows that with increase of* C. polygonoides* ash amount, the % adsorption capacity of MB is increased [52]. This increase in adsorption of MB with the increase of* C. polygonoides* ash amount is due to the increase in the availability of more and more surface area and active sites. Similarly by increasing the surface area more and more adsorption pores are available for MB adsorption [53]. Further increase in concentration of both* C. polygonoides* ash beyond 100 mg the adsorption of MB cannot increase; this may be due to saturation of vacant spaces or the aggregation/agglomeration of biosorbent particles with each other. In the present study therefore 100 mg of* C. polygonoides* ash was optimized. Similar results have been reported by other researchers for other dye adsorptions [54].

### 4.4. Kinetics of Adsorption and Model Fittings

**Figure 8:**
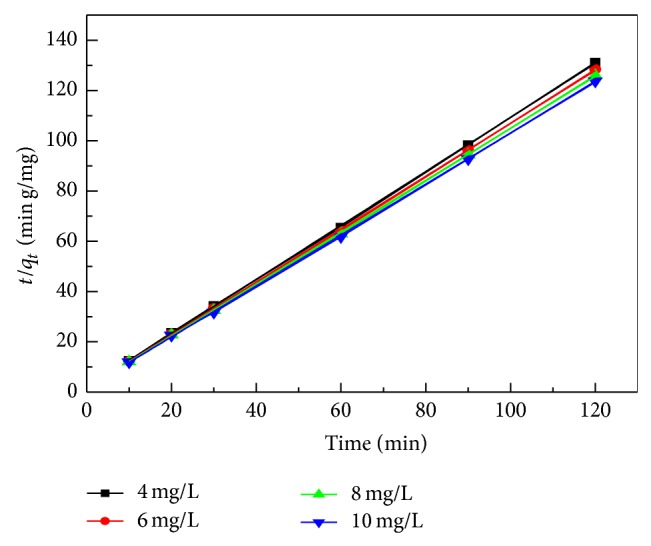
Pseudo-second-order kinetics plot for adsorption of MB on* C. polygonoides* ash.

The adsorption mechanism process of MB on* C. polygonoides* ash was evaluated by using pseudo-first-order and pseudo-second-order kinetic models [55] (Table 4). From Figure 8 and Tables 5 and 6 it is clear that the adsorption of MB on* C. polygonoides* can be fitted in the pseudo-second-order kinetics with regression coefficient (*R* ^2^) 0.999.

### 4.5. Intraparticle and Liquid Film Diffusion Model

The diffusivity of the solute molecules plays an important role in the determination of overall rate of an adsorption process. Although the pseudo-second-order equation was found to the best fitted order for present experimental data, the results obtained from this model are not sufficient to predict the diffusion mechanism. During a solid/liquid adsorption process, adsorbate transfer was usually governed by liquid phase mass transport (boundary-layer diffusion), or intraparticle mass diffusion, or both. The slowest step, which might be either film diffusion or pore diffusion, would obviously be the overall rate-controlling step of the adsorption process. The intraparticle and liquid film diffusion models are represented by

(8) $$\begin{array}{c} q_{t}=K_{i}\cdot dt^{0.5}. \end{array}$$

**Figure 9:**
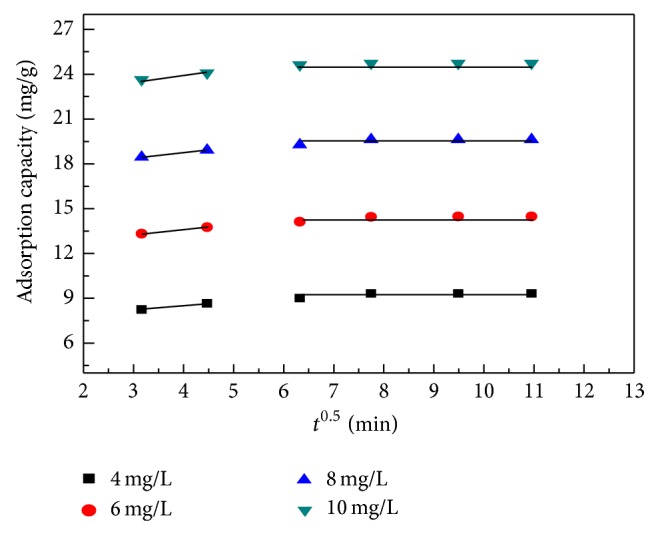
Plot of intraparticle diffusion model for MB adsorption by* C. polygonoides* ash at different initial dye concentrations.

Figure 9 shows the interparticle diffusion model fitting for the adsorption of various concentration of MB. The result shows that the plot of *q* versus *t* ^0.5^ is not very linear for whole time and can be divided into two regions. The multilinearity of the plot for MB adsorption shows the multistage adsorption of MB on the* C. polygonoides*. Normally if the plot of *q*_*t*_ versus *t* ^0.5^ passed through origin this indicates the interparticle diffusion is only the rate limiting step. However in this study it is clear that the plot of *q* versus *t* ^0.5^ is not passing through the origin, which shows that the intraparticle diffusion is not involved in the adsorption process; therefore it is not a sole rate-controlling step. This result confirms that the adsorption process is followed by two or more than two phases [4, 56].

In the first region the plot is linear due to mass transfer, which allows the MB molecules to be transported to the external surface of biomass through film diffusion. In this portion the adsorption is very first because of the strong interaction between the MB molecules and the external surface of the adsorbent.

After boundary-layer diffusion, the MB molecules entered through pores to the interior of the biomass by intraparticle diffusion, as reflected by the second linear portion of the plot. The stage of gradual adsorption whose rate was controlled by intraparticle diffusion was followed by a final equilibrium stage during which the intraparticle diffusion started to slow and become stagnant as the adsorbate molecules occupied all of the active sites of the adsorbent and the maximum adsorption was attained. Although all of the intraparticle diffusion plots for different initial dye concentrations provided a linear relationship, none of the line segments passed through the origin. The nonzero intercepts of the plots indicate that intraparticle diffusion is involved in the adsorption process but is not the sole rate-controlling step for the adsorption of MB. The increment of *k*_*id*_ with increasing initial MB concentration simplifies that the increased driving force at high initial concentrations promotes intraparticle diffusion of the adsorbate onto the adsorbent.

### 4.6. Adsorption Mechanism of Dye

The adsorption mechanism depends on the structure of dye molecule and also the surface of adsorbent. The MB is cationic dye having amine group in its structure formula and while dissolving in water its molecule dissociated into MB^+^ and Cl^−1^. The FTIR result shows that the* C. polygonoides* ash has –OH group which is more exposed and has strong chemical interaction between dye ions and adsorbent and interacts mechanically because of easy penetration of dye ions to the microstructure of adsorbent.

The mechanism for adsorption of MB on adsorbent may be involving the following major steps.

*(a) Migration of Dye Molecules.* In this step the passage of MB molecules from bulk of the solution occurred to the surface of the adsorbent. 

*(b) Diffusion of Dye Molecules*. In this step the diffusion of dye molecules can take place through the boundary layer to the surface of the adsorbent. 

**Figure 10:**
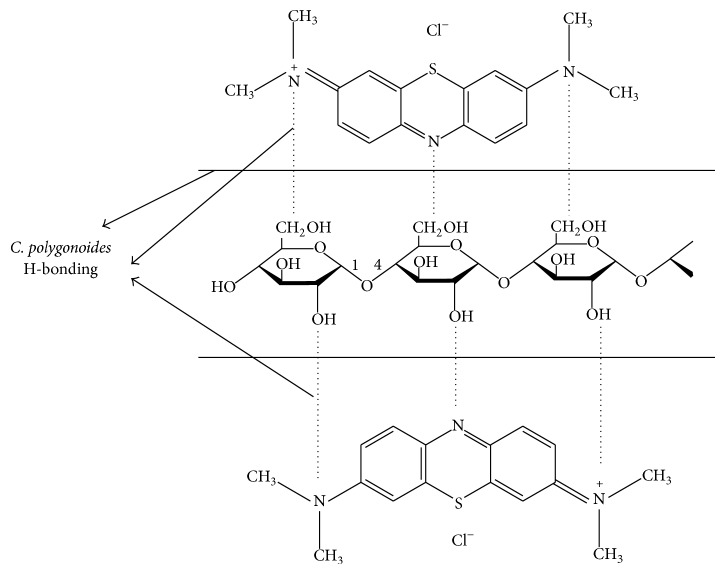
Mechanism of adsorption of MB molecules on biosorbent.

*(c) Adsorption of Dye Molecules.* In this step there is formation of surface hydrogen bonding between the nitrogen atom of MB and hydroxyl group of* C. polygonoides* ash.  Figure 10 shows the proposed possible mechanism for adsorption of MB molecules on the surface of* C. polygonoides* ash

(9) CP–OH⟶CP–O−+H+CP–O−+MB±⟶CP–O–MB+H+

*(d) Intraparticle Diffusion of Dye*. In this final step the dye molecules get approached inside the pores of adsorbent.

## 5. Conclusions

From this present research work the following main conclusions are investigated.

*C. polygonoides* ash is proved to be the promising biosorbent for removal of MB from aqueous solution. The percentage of removal of dye on these biosorbents is fast and adsorption equilibrium is achieved in about 60 min. More than 97% removal of MB was approached in first 60 min after that no increase in adsorption was taking place. Dye adsorption increased with the increase in the amount of adsorbate. The adsorption of MB on* C. polygonoides* ash adsorbent followed the pseudo-second-order kinetics models which are indicated by correlation coefficient values. A comparison of adsorption capacity of* C. polygonoides* ash with many other biosorbents reported in the literature indicates far better performance of* C. polygonoides.* Due to its low cost, high adsorption capacity, and environmentally friendly nature,* C. polygonoides* ash could be used as a promising adsorbent in future for effective large scale removal of MB from aqueous solution on a large scale.

## Supplementary Material

Figure 1: Particle size analysis.
Figure 2: Surface area (BET).

## Figures and Tables

**Table 1:** Resonance structure and some important physical properties of MB (3, 7-bis(dimethyl amino)phenothiazine-5-ium chloride) [30].

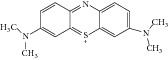		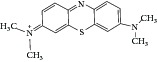		
Mw (g/mol)	Size (nm)	*S* (g/L)	log⁡*K*_ow_	*T*_*m*_ (°C)
319.85	1.69 × 0.74 × 0.38	43.60	5.58	100–110

Mw molecular weight.

*S* water solubility at 25°C.

*K*_ow_ octanol water partition coefficient.

*T*_*m*_ melting point.

**Table 2:** Surface properties of *C. polygonoides* ash.

Surface properties	Values
BET surface area	4.3810 m^2^·g^−1^
Average pores volume	0.016691 cm^3^·g^−1^
Average pore diameter	152.56 Å

**(a):** 

Adsorbent	Shaking time/min	References
*C. polygonoides *	60 min	This work
Tea waste	720	[31]
Jackfruit leaf	300	[32]
Pomelo skin	315	[33]
Jackfruit peel	180	[34]
Coconut bunch waste	300	[35]

**(b):** 

Adsorbent	Adsorption capacity	References
	(mg/g)	
*C. polygonoides *	24.72	This work
NaOH treated raw kaolin	16.34	[36]
NaOH treated pure kaolin	20.49	[36]
Beech sawdust pretreated with CaCl_2_	13.02	[37]
*Calotropisprocera* leaf	4.17	[38]
Orange peel	14.3	[39]
Jute fiber carbon	27.99	[40]
Sawdust	37.83	[41]

**Table 4:** Pseudo-first-order kinetics data for adsorption of MB on *C. polygonoides* ash.

Conc.: mg/L	Intercept	Slope	*R* ^2^
4	−0.711	−0.0220	0.99953
6	−0.6242	−0.0272	0.9839
8	−0.5695	−0.0298	0.9522
10	−0.4653	−0.0361	0.8851

**Table 5:** Pseudo-second-order kinetics data for adsorption of MB *C. polygonoides* ash.

Conc.: mg/L	Intercept	Slope	*R* ^2^
4	1.68	1.07	0.9998
6	1.75	1.05	0.9999
8	1.73	1.03	0.9998
10	1.67	1.01	0.999

**Table 6:** Pseudo-second-order kinetic parameters of various kinetics parameters for the adsorption of MB onto* C. polygonoides *at different initial dye concentrations.

Conc.:	*q*_2_	*k*_2_	*h*	*R* ^2^
mg/L	(mg g^−1^)	(g mg^−1^ min^−1^)	(mg g^−1^ min^−1^)	
4	0.93	0.46	0.402	0.9999
6	0.95	0.51	0.467	0.9999
8	0.96	0.54	0.507	0.9998
10	0.98	0.58	0.567	0.9999
