# Supplementary material for: Potential Biosorbent Derived from Calligonum polygonoides for Removal of Methylene Blue Dye from Aqueous Solution
Source: ScientificWorldJournal. 2015 Jan 15;2015:562693. doi: 10.1155/2015/562693 (PMC4329226; doi:10.1155/2015/562693)
Supplement: Supplementary file 1 — Figure 1: Particle size analysis. Figure 2: Surface area (BET). [file 562693.f1.pdf]

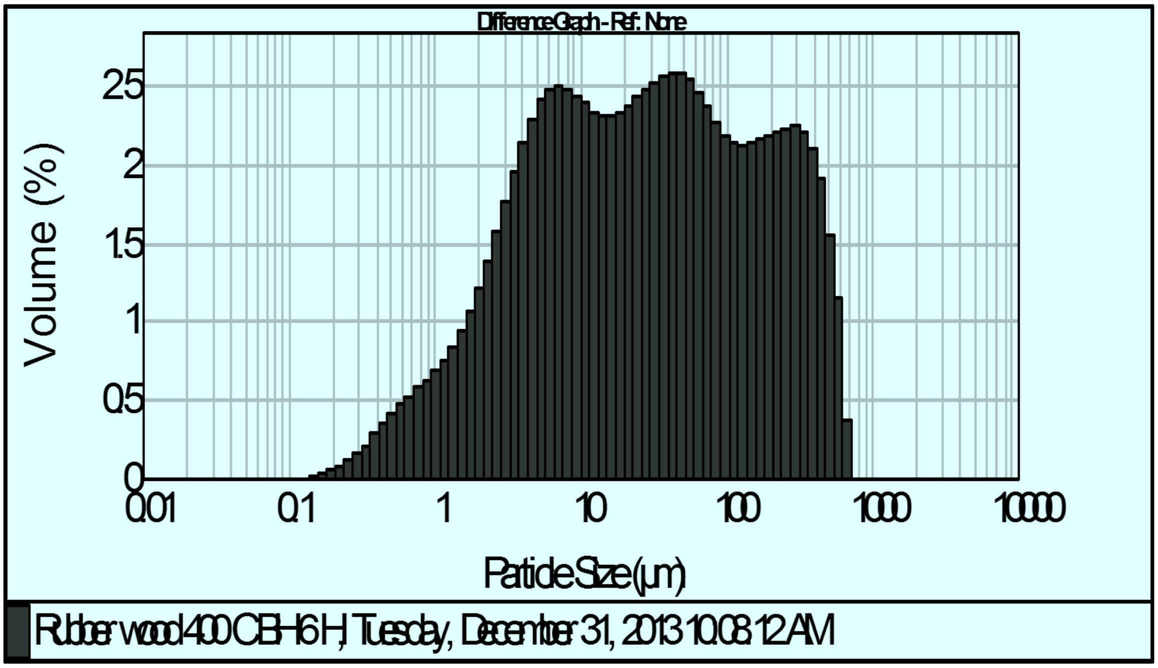

Fig. 1. Particle size of *C. pollygonides* ash.

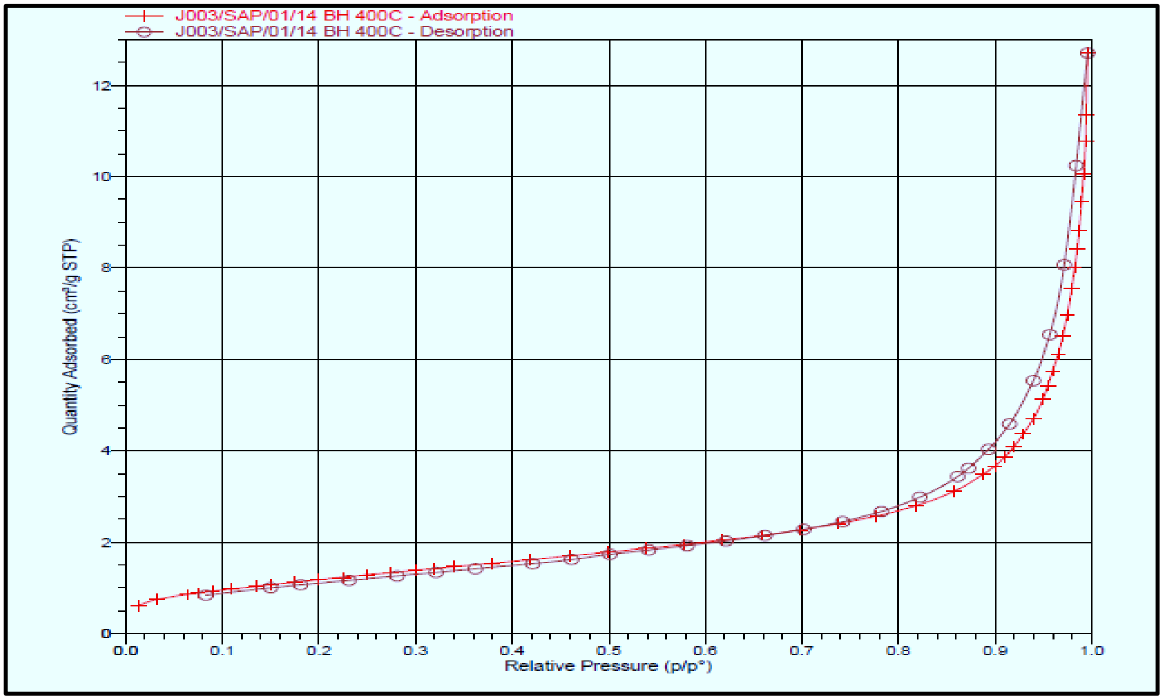

Fig. 2. N_2 adsorption-desorption isotherm of *C. pollygonides* ash
